# The Pandemic Response Commons

**DOI:** 10.1093/jamiaopen/ooae025

**Published:** 2024-04-11

**Authors:** Matthew Trunnell, Casey Frankenberger, Bala Hota, Troy Hughes, Plamen Martinov, Urmila Ravichandran, Nirav S Shah, Robert L Grossman, Pandemic Response Commons Consortium, Casey A Frankenberger, Robert L Grossman, Bala Hota, Troy Hughes, Gina R Kuffel, Plamen Martinov, Pauline Ribeyre, Lea Savatore, Nirav Shah, Eric S Swirsky, Matthew Trunnell, Jacob Krive, Tim Holper, Pamela T Roesch, Nirav Shah, J Alan Simmons, Eric Swirsky, L Philip Schumm, Kenneth J Locey, Robert L Grossman, Zhenyu Zhang, Mihai Giurcanu, Suzet McKinney, Stephanie D Willding, Kim Jay, Pamela T Roesch, Eric Swirsky, Lea Salvatore, Robert L Grossman, Michelle B Hoffman, Keith T Gagnon, Koushik Sinha, Matthew Trunnell

**Affiliations:** Open Commons Consortium, Chicago, IL 60607, United States; Rush University Medical Center, Chicago, IL 60612, United States; Rush University Medical Center, Chicago, IL 60612, United States; Center for Translational Data Science, University of Chicago, Chicago, IL 60615, United States; Open Commons Consortium, Chicago, IL 60607, United States; NorthShore University HealthSystem, Evanston, IL 60201, United States; NorthShore University HealthSystem, Evanston, IL 60201, United States; Center for Translational Data Science, University of Chicago, Chicago, IL 60615, United States

**Keywords:** data commons, regional data commons, data sharing, data systems, pandemic

## Abstract

**Objectives:**

A data commons is a software platform for managing, curating, analyzing, and sharing data with a community. The Pandemic Response Commons (PRC) is a data commons designed to provide a data platform for researchers studying an epidemic or pandemic.

**Methods:**

The PRC was developed using the open source Gen3 data platform and is based upon consortium, data, and platform agreements developed by the not-for-profit Open Commons Consortium. A formal consortium of Chicagoland area organizations was formed to develop and operate the PRC.

**Results:**

The consortium developed a general PRC and an instance of it for the Chicagoland region called the Chicagoland COVID-19 Commons. A Gen3 data platform was set up and operated with policies, procedures, and controls for a NIST SP 800-53 revision 4 Moderate system. A consensus data model for the commons was developed, and a variety of datasets were curated, harmonized and ingested, including statistical summary data about COVID cases, patient level clinical data, and SARS-CoV-2 viral variant data.

**Discussion and conclusions:**

Given the various legal and data agreements required to operate a data commons, a PRC is designed to be in place and operating at a low level prior to the occurrence of an epidemic, with the activities increasing as required during an epidemic. A regional instance of a PRC can also be part of a broader data ecosystem or data mesh consisting of multiple regional commons supporting pandemic response through sharing regional data.

## Background and significance

**Figure 1.**
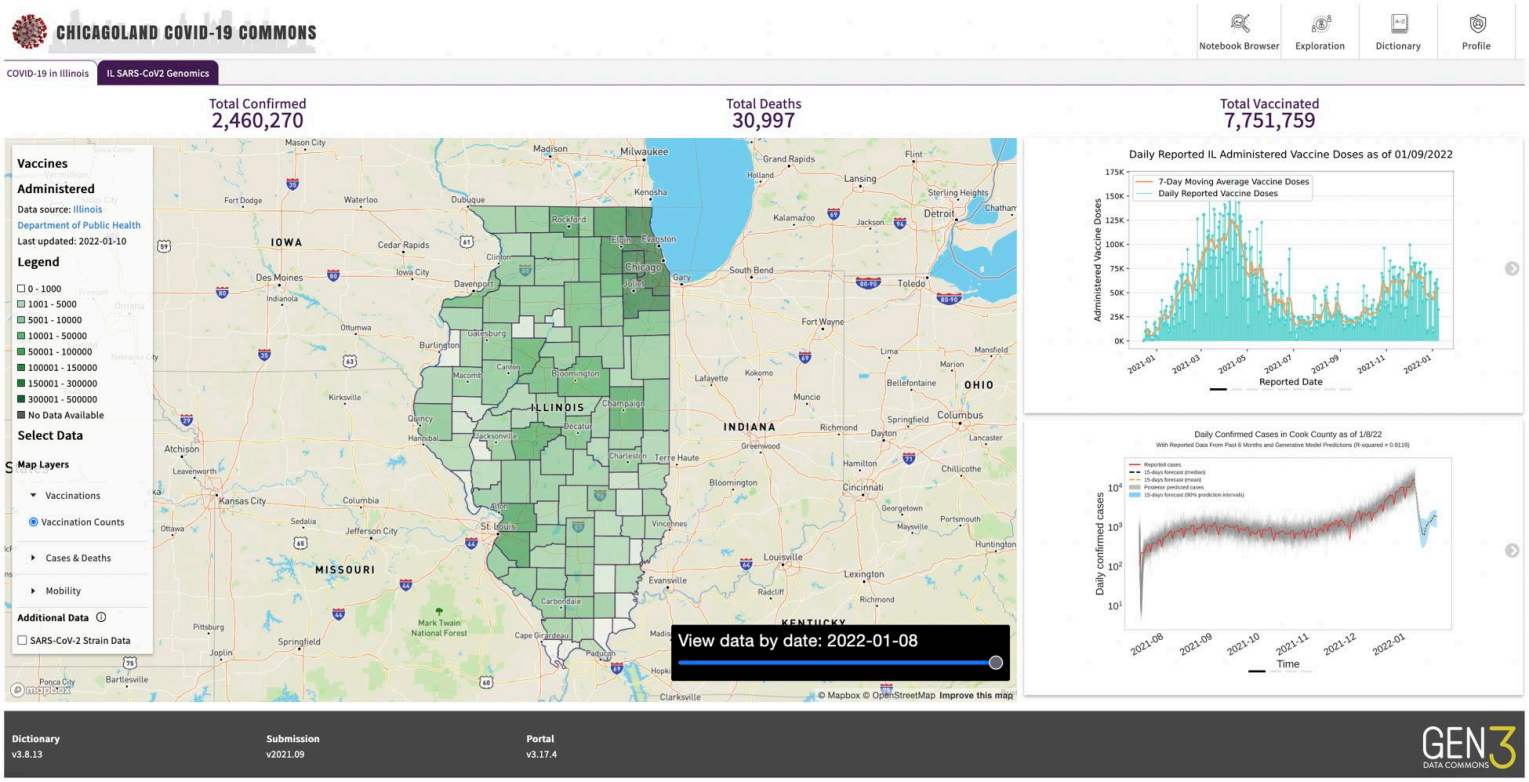
A screenshot of the Chicagoland COVID-19 Commons.

A data commons is a software platform for managing, harmonizing, analyzing, and sharing data with a community.^1^ The Pandemic Response Commons (PRC) is an open source data platform for rapidly setting up data commons for a consortium of researchers, public health workers, and decision makers in response to an epidemic or pandemic. In particular, the Chicagoland COVID-19 Commons (CCC) is an instance of the PRC that serves the Chicago region, and, more broadly, the state of Illinois and surrounding regions. See Figure 1.

The CCC contains clinical data from about 135 000 subjects, over 6600 viral genomes, and public data from a variety of sources.

There were many efforts to collect COVID-related data since the start of the COVID-19 pandemic. These include required data collection as part of local and state public health reporting, required data collection as required by federal statutes and regulations, including by the Center for Disease Control (CDC), research-related efforts by various NIH Institutes and Centers,^2^ and volunteer efforts. Important projects that provided COVID-19-related data include projects by the John Hopkins University Center for Systems Science and Engineering, the New York Times, and the COVID Tracking Project. Data were also released by companies, for example, the mobility data collected, processed, and released by Google. Today, there are many NIH supported projects that are collecting COVID and Long COVID-related data, including the NIH Post-Acute Sequelae of SARS-CoV-2 infection (PASC) Project and the National COVID Cohort Collaborative (N3C).^2^

An important role of a data commons is to curate, integrate, and harmonize data for a specific community, generally for research purposes. As can be seen from the COVID Tracking Project^3^ and the CDC COVID Tracking Data,^4^ there are important regional differences in the incidence, fatalities, and health disparities associated with COVID-19. This suggests the utility in collecting, integrating, and analyzing COVID-19-related data at the local and regional levels, and then aggregating the results. The PRC is a data platform designed to support this type of local, regional, and federated analysis.

## Methods

### Consortium governance

The Pandemic Response Consortium (Consortium) is a private-public partnership that is operated by the Open Commons Consortium (OCC), part of an independent 501(c)(3) not-for-profit corporation that operates several other data commons, including the BloodPAC Data Commons^5^ and the Veterans Precision Oncology Data Commons.^6^

The Open Commons Consortium provides templates for Consortium Membership Agreements, Data Contributors Agreements, Data Use Agreements, Intellectual Property Agreements, and related agreements so that: (1) a consortium can be established to build and operate a data commons, (2) data can be contributed to a data commons, and (3) members of the consortium can form working groups to analyze data in the commons and develop applications, notebooks, and software services to enhance the functionality of the commons.

Currently, the PRC has 4 working groups: the Clinical Data Working Group, the Epidemiological Modeling Working Group, the Community Engagement and Outreach Working Group, and the Data Commons Working Group.

### Data governance

Consortium members that contributed clinical data first obtained IRB approval. The IRBs submitted by members were based upon a common template developed by the consortium. The Clinical Data Working Group met biweekly for several months to develop and refine the data model that each consortium member contributing data used to provide data.

In addition to clinical data, the CCC collected several other types of data from CCC Members, including aggregate counts for different types COVID-related incidences, and sequencing data about viral variants. The CCC also collected public data of interest to the COVID research community, including case and fatality data, mobility data, public clinical, and imaging data about COVID cases.

### Data model

A Gen3 data commons is auto-generated from a graph data model and this process produces FAIR APIs^7^ that can be used to access the structured data in the commons, as well as the data objects and their associated metadata. For the CCC, the graph data model is a graph with 40 nodes and over 1200 data attributes. The data model can be viewed in a graphical and tabular format in the CCC portal (https://chicagoland.pandemicresponsecommons.org/dd) and the properties, including definitions and allowed values, associated with individual graph nodes can be downloaded in JSON and TSV formats. The model is broadly based upon the data model used by the NCI Genomic Data Commons^8^ and the nodes in the graph are grouped into several categories including biospecimen nodes, such as sample and aliquot nodes; clinical nodes, such as observation, follow up, demographic and summary nodes; molecular data nodes, such as virus sequence files, virus sequence alignment files, and virus sequence contig files; data file nodes, such as imaging and index file nodes; and, administrative nodes, such as project, study, and location nodes. Only a few nodes are required, such as the project node, and each commons develops their own data model. For the CCC, the Clinical Data Working Group developed a data model for COVID-related clinical symptoms and the Variant Surveillance Working Group developed nodes to support SARS-COV-2 sequence data.

### Clinical data and its harmonization

The Clinical Data Working Group developed a data model to support data modeling efforts with a particular focus on disparities and comorbidities. Adapted from the CDC case report form for persons under investigation developed early in the pandemic, the requested data elements include demographics, symptoms, comorbidities, and lab results. In addition, basic information about a patient’s encounter was requested, including whether the patient was admitted to the hospital, whether they were transferred to the ICU, and whether they were placed on a ventilator and for how long.

As mentioned above, the data model is a graph data model with 40 nodes and data attributes (called properties) attached to the nodes. There are 3 main graph nodes summarizing patient level clinical data: the Demographic node with 17 attributes, the Observation node with 95 attributes, and the Follow Up node with 14 attributes, for a total of 126 clinical data attributes.

The Clinical Data Working Group had to agree on precise definitions of a COVID patient (based on results from one or more of a specified set of laboratory tests) and COVID encounter, defined as a hospital visit within 30 days of a positive COVID test. The definitions used internally by the healthcare organizations varied significantly across the partners. The group also had to agree on precise lists of clinical data codes (ICD-10 codes) describing comorbidities and laboratory results.

The requested data include race and ethnicity where available. This allows questions of disparities in outcomes and variation in occurrence of symptoms and comorbidities to be studied. These data elements are being reported quarterly by participating healthcare partners for all COVID patients.

### Infrastructure for modeling

The Gen3 data platform is based upon Kubernetes and Kubernetes is used for bringing up Docker containers with computational tools and workflows for modeling. Data for modeling are available through the Gen3 APIs for both structured data from the Gen3 graph data model and data objects from the Gen3 object storage (https://gen3.org). Access to the Internet is restricted and any needed public data are imported to Gen3 or the website is added to a white list of external sites that can be accessed by Gen3 workflows. In addition, Jupyter notebooks that could access data in the CCC were provided to members of the Consortium analyzing the data.

### Security and compliance

The PRC follows the policies, procedures, and controls for a NIST SP 800-53 revision 4 Moderate system.^9^ It also undergoes a yearly penetration test, and a yearly security and compliance audit by a third party.

## Results

### Consortium membership

Currently, the Chicagoland instance of the Pandemic Response Commons Consortium includes 8 members, each of which has signed consortium membership agreements. The consortium members are: Rush University Medical Center, NorthShore University HealthSystem, University of Chicago, University of Illinois at Chicago, St Anthony Hospital, Sinai Chicago, CommunityHealth, and Southern Illinois University.

Rush University Medical Center, Northshore University HealthSystem, the University of Chicago, the University of Illinois at Chicago, Sinai Health System, St Anthony Hospital, and others have contributed data to the Chicagoland instance of the PRC. In total, the CCC contains clinical data from over 135 000 subjects with COVID. All controlled access data were contributed under the CCC Data Contributors Agreement under an IRB or with an IRB exemption and can be shared with other consortium members, and, in aggregated form, with other researchers.

### Working group activities

CCC activities are organized into 5 working groups, each of which has a charter. These were the Clinical Data Working Group, the Epidemiological Modeling Working Group, the Community Engagement Working Group, the Variant Surveillance Working Group, and the Commons Operations Working Group.

The Clinical Data Working Group developed a common data model for the commons and worked with each member to contribute data in the required format. In addition to clinical data, the CCC also collected aggregated counts for cases, deaths, and selected comorbidities that are used by the Epidemiological Modeling Working Group to understand health disparities and to build predictive models.

The Epidemiological Modeling Working Group initially developed hierarchical Bayesian models that predicted future COVID case and fatality counts by county in Illinois. The models were adapted from the models developed by Imperial College.^10^ These models were updated weekly. The Epidemiological Modeling Working Group also developed a generative Bayesian for the Chicago region that was run daily. The Working Group has also developed regression models to understand race/ethnicity differences in case/fatality ratios based on temporal and age-related factors. These models are described in greater detail below.

The Variant Surveillance Working Group has collected over 6632 SARS-CoV-2 and contributed them to the CCC, as well as to other national and international virus variant databases.

### Pandemic Response Commons

As mentioned, the PRC was developed with the open source Gen3 software platform (https://gen3.org/). Once the Data Working Group defines a data model or updates an existing data model, the Gen3 software platform automatically generates a data commons with APIs for data access, metadata access, data submission, and authorization and authentication. In this way, the data commons protects controlled access data and makes both controlled access and open access data findable, accessible, interoperable, and reusable (FAIR).^7^

Currently, there is one regional instance of the PRC (https://pandemicresponsecommons.org)—the CCC. An instance of the PRC includes a portal for exploring, submitting data, and browsing publicly accessible PRC Jupyter Notebooks. The PRC Notebook Browser, which is available from the PRC portal, includes over a dozen Jupyter notebooks that are built over data from the PRC, including case and fatality data, open access clinical data, mobility data, and COVID-19 imaging data.

### Patient level data for COVID-19

To date, 5 institutions (NorthShore University HealthSystem, Rush University Medical Center, University of Chicago Medical Center, University of Illinois at Chicago Health, and Sinai Chicago) have submitted Patient Summary Reports with patient-level data starting from March 1, 2020. The CCC has focused on analyzing submitted information for validation and to identify data quality issues, such as differences in format and missing data. The Data Commons Working Group developed a process with each organization in the CCC for submitting data. The Working Group also developed custom Python code as required so that the data submitted could be converted to the proper format and uploaded and ingested by the commons. In addition, the Data Commons Working Group ran Python-based data quality checks to discover simple formatting and other errors prior to ingesting the data.

Further quality analysis of the uploaded data included developing plots that compared patient counts by demographic characteristics, presenting symptoms, events (ventilation, ICU admission, etc.), comorbidities, and complications. Table 1 shows the number of cases (over 135 000) provided to the PRC. The counts in Table 1 should be considered approximate. In particular, it is important to note that institutions identify COVID patients that are contributed to the PRC based on a rule (evidence of positive test result within 30 days of admission), while counts of COVID patients reported for other purposes would in general use different criteria.

**Table 1.** Patient level data in the Chicagoland COVID-19 Commons.

Institution	Number of patients in submission
NorthShore University HealthSystem	26 829
Rush University Medical Center	39 420
University of Chicago Medical Center	24 076
University of Illinois at Chicago Health	28 721
Sinai Chicago	16 476
Total	135 522

### Patient level data for long COVID

In 2022, the Clinical Data Working Group and the Epidemiological Modeling Working Group worked together to pivot the PRC to study Long COVID. Given the complex nature of long COVID, the decision was made to switch from a feature-based collection of data for each patient to an encounter-based collection of data for each patient. In more detail, when the CCC started collecting COVID-related data, specific features for each patient were collected, such as shortness of breath, cough, headache, nausea or vomiting, and abdominal pain. The response for each feature was limited to a set of possible responses, including unknown. Given the wide variety of clinical conditions associated with Long COVID, this approach was considered to be impractical, and, instead, for each encounter, the following data were collected: encounter type (hospital, ambulatory, telemedicine, medical office), encounter start date, encounter end data, list of ICD-10 codes associated with the encounter, and a list of CPT codes associated with the encounter. In retrospect, it was easier for the healthcare providers to provide this data instead of the feature-based data originally requested, and the data provided were much more useful. On the other hand, collecting encounter-based data placed a greater burden on the Commons Operations Working Group, who had to parse the data for the other working groups to analyze. By January 2023, patient level data on over 26 000 Long COVID patients has been submitted to the CCC. This is expected to grow to over 100 000 Long COVID patients as soon as amended IRBs are approved by the other organizations in the CCC.

### Summary level data

PRC statistical summary reports (SSR) contain aggregate counts in different categories. As aggregate data, they are easier for contributing organizations to share, and, at a sufficiently high level of aggregation, are open access. Count level data from the SSR and other sources are used in various ways by the PRC, including to develop epidemiological models, to provide information for map overlays that are shared with the public, and to provide information for data that is “returned to the community” as part of the activities of the Community Engagement and Outreach Group.

Three institutions (NorthShore University HealthSystem, Rush University Medical Center, and University of Chicago Medical Center) have submitted some form of Statistical Summary Reports of aggregate data. These data were used for building predictive models for case and fatality estimation conditioned on race and ethnicity. In 2022, given the wider availability of this data, the PRC stopped collecting Statistical Summary Reports. Initially, Statistical Summary Reports were designed to be less of a burden and with the expectation that agreements to provide summary counts would be faster to complete. In practice, this was not the case, and thus the decision was made to simply collect Patient Level Data, from which the necessary statistical summary data could be easily computed.

### SARS-CoV-2 variant data

The CCC has been working with Southern Illinois University (SIU) on a project to analyze the genomic sequence of SARS-CoV-2 virus isolated from samples from patients that test positive for COVID.^11^ By studying the distribution of genomic variants over time we can better understand the spread of the disease across the state. In addition, in partnership with Illinois Department of Health (IDPH), we have worked to identify the appearance of specific “Variants of Concern” (VOCs).^12^

**Figure 2.**
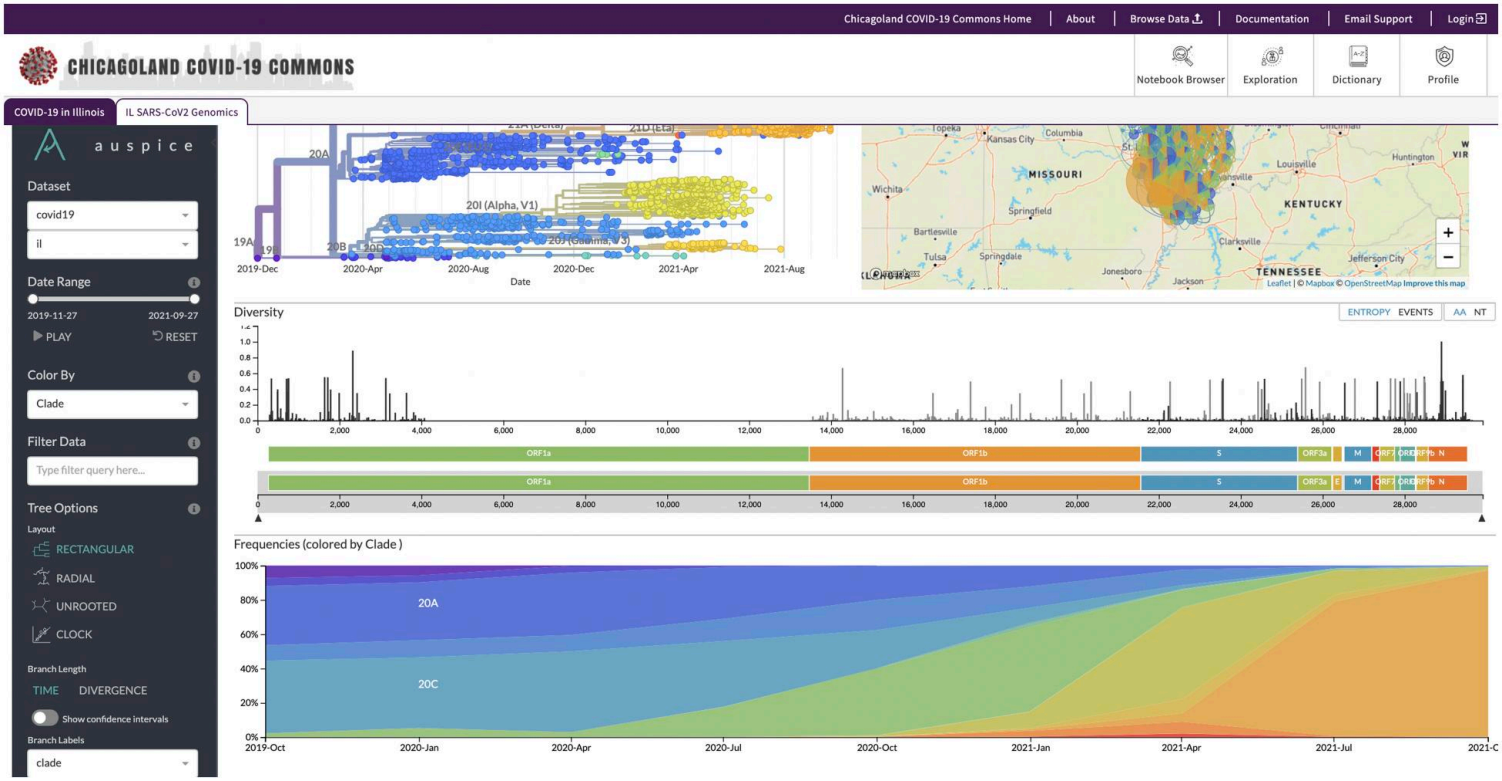
A screenshot of viral variants and their geographic distribution in the Chicagoland COVID-19 Commons.

The project has sequenced over 6632 SARS-CoV-2 genomes over the course of the pandemic. The genomes span 19 viral clades and include more than 150 variants with sufficient resolution to illuminate the evolution of the viral population in Illinois (Figure 2).

### Modeling activities

The CCC Epidemiological Modeling Working Group implemented several models for predicting COVID-19 cases, hospitalizations, and deaths using data from the statistical summary reports provided by Consortium members. The first model the working group implemented was based on the Imperial College Bayesian model^13^ using local data and Google mobility data. Basic SEIR^14^ and generative models were also developed by the working group. In the end, the various models developed by the CCC turned out to be no more useful information than simple regression and curve fitting models for making predictions. In particular, the spline-based models developed by one of the working group members^15^ was simpler, faster, and just as accurate as the other models developed by the CCC. The Bayesian model developed by the working group was very dependent on the Case Fatality Rate (CFR), and understanding the CFR and related health disparities occupied the majority of the Epidemiological Modeling Working Group efforts, leading to the publication.^16^

Table 2 summarizes some of the analysis in Ref.^16^, where the Case Fatality Rate of Black/African Americans (AA) is compared to Whites. Note that at the population level, the CFR for Black/AA is less than the CFR for Whites, but this is reversed at the subgroup level for each of the 3 age groups (18-49, 50-64, and 65+), an example of Simpson’s Paradox.^17^^,^^18^ This reversal between the population level and the age-stratified subgroup level was first seen in the CCC and verified later with state level national data from the CDC, which was then used for the analysis in Ref.^16^, since it was of more general interest.

**Table 2.** Case fatality rate (CFR) ratios comparing minority racial and ethnic groups to Whites,^16^ based upon a mixed effects Poisson regression model described in Ref.^16^

	Black/AA compared to White rate ratio, (95% CI)	Latinx compared to White rate ratio, (95% CI)
Overall (population level)	0.5 (0.4, 0.6)	0.2 (0.2, 0.3)
Ages 18-49 (subgroup level)	6.0 (3.4, 10.8)	4.1 (1.9, 8.7)
Ages 50-64 (subgroup level)	2.7 (1.8, 4.1)	2.4 (1.5, 3.8)
Ages 65+ (subgroup level)	1.0 (0.8, 1.1)	1.2 (1.0, 1.4)

### Educational activities

**Figure 3.**
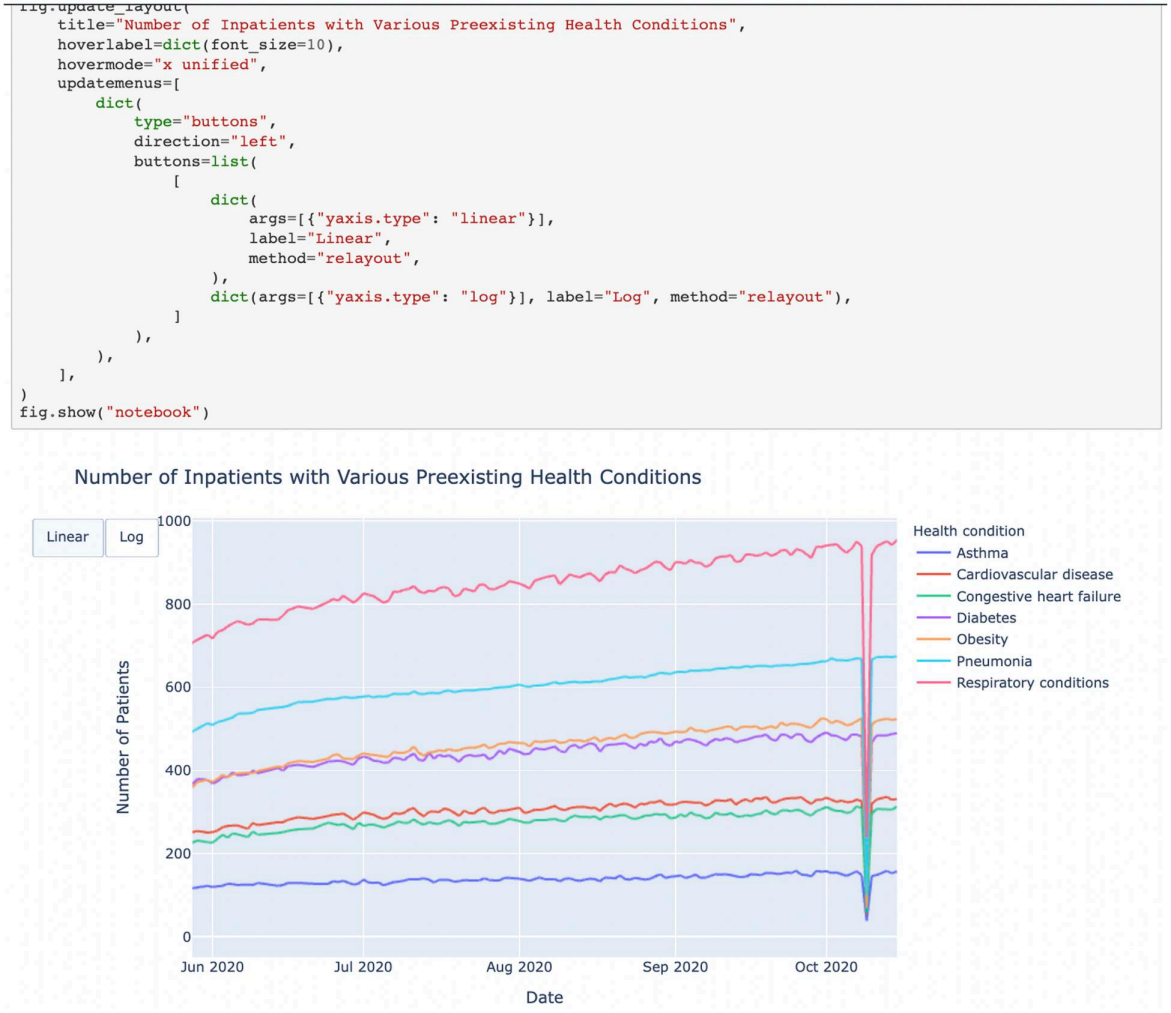
A screenshot of a Jupyter notebook from the Chicagoland COVID-19 Commons.

Educational materials, including Jupyter notebooks, were prepared describing health disparities in COVID-19 and how these varied by race/ethnicity, age group, geographical area, and time period, and how these factors interacted. These tutorials served 2 purposes: first, there was an introductory tutorial that showed how to use Jupyter notebooks and how to access and explore data from the CCC. See Figure 3. Second, there was a tutorial that introduced the topic of health disparities and explained CFR how CFR depends upon age, race/ethnicity, and other factors. The tutorials were recorded and are available from the CCC portal.

## Discussion

### Regional COVID commons

There are several important ongoing efforts to gather data at the national level to support clinical research, policy development, and decision making at the national scale.^2^ To drive regional decision making, a regional COVID commons can be an impactful complement to these national efforts for several reasons. First, local data can be richer, since a regional commons can collect, analyze, and share some data within its own community that might not be so readily available or of interest to a broader community. Second, regional commons can more easily reflect community concerns about data collection and data use compared to national commons. Third, regional commons can potentially make decisions that make sense for a local community, but may not be as relevant or as practical at a national level.

### Interoperating regional COVID commons

Over the last several years, technology has been developed so different data commons can interoperate and share data in ways that are safe and compliant with relevant regulations and policies. Data that are open and unrestricted can be shared directly; data that are sensitive or otherwise restricted might be shared in aggregate or summary form. In this way, a national COVID-19 data common can be developed that brings together some of the data in regional data COVID-19 data commons, while leaving some of the more sensitive data in place. Even with data remaining in place in regional data commons, this information can still be used through a federated analysis, in which an analysis is distributed over the data in each commons, and the results are returned to provide an integrated picture. In this way, data from multiple regional data commons can be used to enhance the national view of COVID-19-related issues.

### The importance of federated learning

Regional, and, more granularly, organizational data commons, provide a mechanism for the collection and analysis of data by those most knowledgeable about it. In addition, they can contribute towards federated learning across multiple commons operated by different organizations and consortia. The Gen3 data commons allow workflows in dockerized containers to be executed locally, with the results reviewed if necessary, and returned for aggregation.

### Preparatory for the next epidemic and pandemic

A large part of the effort in building the PRC and the Pandemic Response Commons Consortium was putting in place all the necessary legal agreements (Consortium Membership Agreements, IRBs, Data Contributor Agreements, Data Use Agreements, Intellectual Property Agreements, etc.) so that members could contribute data to the commons. Now that these legal agreements are in place, the commons can be relatively easily used for monitoring for the emergence of new pathogens, and new epidemics and pandemics if they occur in the future. In other words, infrastructure such as the PRC can be thought of as part of a preparatory infrastructure to make communities more resilient to future outbreaks.

As an example, in 2022, the CCC decided to shift from studying COVID-19 to studying Long COVID. After several months of joint meetings of the Clinical Data Working Group and the Epidemiological Modeling Working Group discussing how best to collect and study Long COVID, the decision was made to move from attribute-based collection of patient level clinical data to encounter-based collection of clinical data once a condition was triggered. In this case, the presence of a positive COVID-19 test or diagnosis in the EMR. Given that all necessary legal agreements were in place, the data commons was in place, the SOPs for transferring data to the data commons were agreed to and the associated training had taken place, the infrastructure for analyzing data in the data commons was in place with the associated training completed, all that was required was to amend the IRB, and change the code used by Clinical Research Data Warehouse for producing the quarterly outputs. As mentioned, data for over 25 000 Long COVID patients is already in the CCC, with over 100 000 Long COVID patients expected by 1Q or 2Q 2023. Just putting in place the correct legal agreements and the SOPs for transferring data took between 1 and 2 years, depending upon the organization, initially.

Importantly, with this new approach of asking for encounter-based data (vs attribute-based data), it is relatively easy to amend the IRB, and add a new condition, when a new public health situation arises. For example, monkeypox data are now being collected from some members in the CCC.

### Engaging with the community

Another important aspect of the CCC is engaging with the community through the Community Engagement and Outreach Working Group. In the words of one of the CCC members, since data are “from the community” it should be “for the community.” Standing up the Community Working Group took some time, given how busy the working group members were during the pandemic. The working group suggested several topics to explore, which included trying to quantify the COVID-related health disparities in the region and providing tutorials to area students so that they could better understand these disparities. This led to publication^16^ described above and to the tutorials that explained how to explore data with Juypter notebooks to understand COVID-related health disparities.

### Challenges and limitations

Putting in place a regional PRC faces 3 main challenges. First, although there are existing legal agreements for consortium membership, data contributors agreements, and data use agreements that have been approved and signed by multiple organizations, as usual, it takes time and effort to put these in place for a new regional commons with new organizations. Second, initial work is required to extract the required data from the organization’s data warehouse or EMR system. Once this is done, producing regular updated data is usually straightforward. Third, keeping a PRC in a preparatory state for a future epidemic or pandemic still requires some low level of IT support from each member organization and for the organization running the commons and a sustainability model is required for these funds.

## Conclusion

The CCC is a data commons for the Chicagoland and Illinois region to accelerate research in COVID and Long COVID. It contains clinical data from over 135 000 patients that have experienced COVID, statistical summary reports supporting the analysis of COVID-19 health disparities, over 6632 COVID-19 viral variants, and a variety of COVID-related public data. Data are available to CCC consortium members and in aggregated form to a broader community. The CCC operates following the policies, procedures, and controls of NIST SP 800-53 revision 4 Moderate system.

The CCC was developed using the open source Gen3 data platform and all the necessary legal agreements to set up a consortium and operate a data commons are available so that other regions can set up their own regional data commons containing their own data. The CCC is also designed with FAIR APIs allowing it to interoperate in a secure and compliant fashion with other regional data commons and other data commons with the necessary security and compliance. In particular, it can be used for distributed and federated machine learning of COVID-related data.

The CCC is designed to provide a long persistent infrastructure that can be “activated” when required in the future to support research around a new epidemic or pandemic. In particular, all the necessary legal agreements and SOPs for contributing data from Consortium members are in place.

## Data Availability

The work described in the article did not generate data nor analyze data but developed and operated a software platform—the Pandemic Response Commons (PRC). Open access data are available through the PRC’s APIs. Computations over the PRC’s controlled access data can be done by submitting Docker containers to the PRC.
